# Cranberries and Cancer: An Update of Preclinical Studies Evaluating the Cancer Inhibitory Potential of Cranberry and Cranberry Derived Constituents

**DOI:** 10.3390/antiox5030027

**Published:** 2016-08-18

**Authors:** Katherine M. Weh, Jennifer Clarke, Laura A. Kresty

**Affiliations:** 1 Department of Medicine, Division of Hematology and Oncology, Medical College of Wisconsin, 8701 Watertown Plank Road, Milwaukee, WI 53226, USA; kweh@mcw.edu; 2 Department of Food Science and Technology, University of Nebraska, 256 Food Innovation Complex, Lincoln, NE 68588-6205, USA; jclarke3@unl.edu; 3 Department of Statistics, University of Nebraska, Lincoln, NE 68583, USA; 4 Quantitative Life Sciences Initiative, University of Nebraska, Lincoln, NE 68583, USA

**Keywords:** cranberry, cancer, proanthocyanidin, quercetin, ursolic acid

## Abstract

Cranberries are rich in bioactive constituents reported to influence a variety of health benefits, ranging from improved immune function and decreased infections to reduced cardiovascular disease and more recently cancer inhibition. A review of cranberry research targeting cancer revealed positive effects of cranberries or cranberry derived constituents against 17 different cancers utilizing a variety of in vitro techniques, whereas in vivo studies supported the inhibitory action of cranberries toward cancers of the esophagus, stomach, colon, bladder, prostate, glioblastoma and lymphoma. Mechanisms of cranberry-linked cancer inhibition include cellular death induction via apoptosis, necrosis and autophagy; reduction of cellular proliferation; alterations in reactive oxygen species; and modification of cytokine and signal transduction pathways. Given the emerging positive preclinical effects of cranberries, future clinical directions targeting cancer or premalignancy in high risk cohorts should be considered.

## 1. Introduction

Incorporation of fruit and vegetables, including cranberries, into a healthy-balanced diet is suggested for prevention of human disease. The positive health benefits of cranberries and cranberry derived constituents include improvements of cardiovascular function as measured by decreases in lipid peroxidation, oxidative stress, total and low-density lipoprotein (LDL} cholesterol and high-density lipoprotein (HDL) cholesterol level increases [1,2]. Cranberry derived products can also increase immune function by increasing γδ-T cells, NK cells and B-cells [3], as well as exhibit antimicrobial and anti-adhesion activities against Gram-positive bacteria [4], Gram-negative bacteria [5,6,7,8,9,10,11] and yeast [12,13]. Utilization of cranberry and cranberry derived constituents in the prevention of cancer is an underexplored area, but one with mounting preclinical in vitro and in vivo research as will be reviewed herein. To date, there have been no clinical trials conducted which utilize cranberries to prevent or delay cancer progression.

The beneficial effects of cranberries are attributable to the berries’ rich phytonutrient composition which has been extensively and expertly reviewed by Pappas et al. [14]. Compositional analysis of the cranberry has resulted in identification and characterization of over 150 different bioactive constituents and human metabolomic studies have revealed differential pharmacokinetic profiles for these molecules [14,15,16,17]. Included in the family of polyphenols are three flavonoid classes: anthocyanins, flavonols and proanthocyanidins. Specifically, flavonoids and phenolic acids are detected in the urine and plasma of healthy older adults following a single dose of 54% cranberry juice [15]. Cranberries and cranberry derived constituents are capable of exerting antioxidant and anti-inflammatory functions as supported by several clinical trials investigating cardiovascular health improvements measured via increases in flow mediated dilation, total antioxidant performance of plasma, blood glutathione peroxidase levels and superoxide dismutase activity following consumption of a cranberry juice cocktail [15,18,19,20,21].

**Figure 1:**
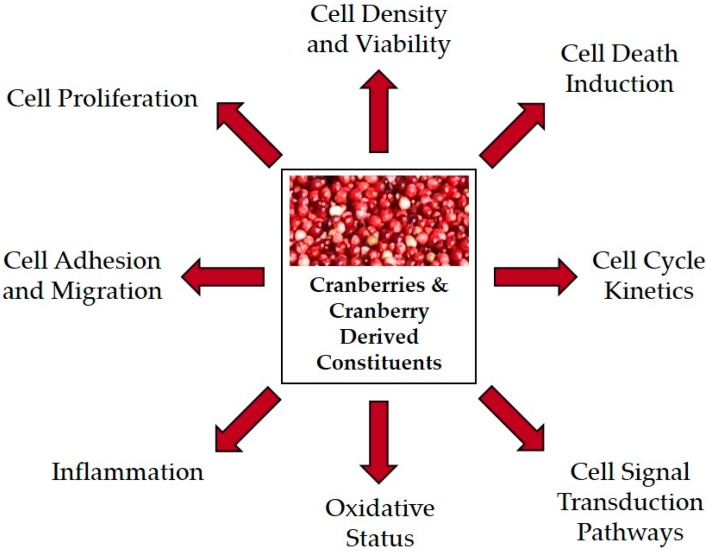
Cranberry and cranberry derived constituents target numerous mechanisms of cancer inhibition based on 34 preclinical studies.

The cancer inhibitory potential of cranberries and cranberry derived products is being elucidated based on multiple in vitro investigations and a small number of in vivo studies. This review will encompass a total of 34 preclinical studies utilizing 45 cancer cell lines isolated from 16 target organs and studies targeting seven cancers utilizing in vivo carcinogenesis and xenograft models to investigate mechanisms by which cranberries and cranberry derived constituents modulate or inhibit cancer-related processes. Mechanisms of cranberry-linked cancer inhibition are summarized in Figure 1. Preclinical studies support that cranberries modulate cell viability, cell proliferation, cell death, adhesion, inflammation, oxidative stress and signal transduction pathways. Many of the in vitro studies initially focus on the effectiveness of cranberry derived constituents in cell density and viability assays, as logical starting points for determining whether further mechanistic analysis is warranted. Collectively, these in vitro studies provide the fundamental basis for additional in vivo studies and may inform the design and implementation of cancer-based clinical trials evaluating cranberries as cancer preventive agents.

## 2. Materials and Methods

A thorough bibliographic search was conducted in Pubmed through 4 June 2016 to identify all cancer focused research utilizing cranberries or cranberry derivatives. Keyword searches were performed by searching cranberry and each individual cancer target: breast, cervical, colon, esophageal, glioblastoma, leukemia, liver cancer, lung, lymphoma, melanoma, neuroblastoma, oral cavity, ovarian, prostate, renal/kidney, stomach and bladder. A secondary search was conducted using the same keywords in Scopus, an abstract and citation database for peer-reviewed literature, which yielded additional manuscripts not available in Pubmed. Finally, a bibliographic search was completed in the Health Research Library provided by the Cranberry Institute using the following keywords: cancer, reactive oxygen species, anti-oxidant and oxidative stress.

## 3. In Vitro Inhibition of Cancer Processes by Cranberries

Utilization of cancer cell lines to test cranberries, cranberry derived constituents and extracts has been the initial basis for defining cancer inhibitory capacity of these natural components in vitro. The majority of studies have been performed following treatment of immortalized cancer cell lines with cranberry extracts or juices, while one study has determined the benefit of pretreating the cells first to measure protective capacity against oxidative stress [22]. As summarized in Table 1, there are 31 in vitro based published reports describing cranberry linked cancer inhibition in 45 cancer cell lines derived from 16 targets. Eight mechanisms will be discussed with respect to cranberry derived extracts and constituents including cell density and viability, cell proliferation, cell cycle kinetics, cell death, signaling pathways, adhesion and migration, oxidative status and inflammation.

### 3.1. Cranberry Derived Extracts and Constituents Affect Cellular Growth and Viability

A significant amount of research shows cranberry derived constituents decrease cancer cell density, viability and proliferation. Cell density experiments are primarily based on treatment of cell lines with cranberry derived extracts followed by crystal violet staining to visualize cells remaining after treatment. While crystal violet staining indicates a qualitative difference in cellular confluency based on treatment, it does not rely on active metabolic processes. Viability stains including 3-(4,5-dimethylthiazol-2-yl)-2,5-diphenyltetrazolium bromide (MTT) and 2-(4-iodophenyl)-3-(4-nitrophenyl)-5-(2,4-disulfophenyl)-2H-tetrazolium, monosodium salt (WST-1) rely on cleavage of tetrazolium salts by the succinate-tetrazolium reductase system in metabolically active cells with an intact mitochondrial respiratory chain, whereas Calcein-acetoxymethyl (Calcein-AM) is cleaved by intracellular esterases to a fluorescent molecule, which results in more reliable data when cell death involves altered mitochondrial machinery. Bromodeoxyuridine (BrdU) incorporation assays as a measurement of cell proliferation inform how cranberry derived constituents modulate S-phase cell cycle kinetics.

Three cranberry derivatives inhibit the growth of 43 human cancer cell lines. For the purpose of this review, growth inhibitory (GI_50_) concentrations are presented for cancer cell lines where cranberry treatment results in 50% growth inhibition. Specifically, cranberry derived ursolic acid, proanthocyanidins and an organic-soluble cranberry extract inhibit the growth of breast, colon, cervical, glioblastoma, leukemia, lung, melanoma, oral cavity, prostate and renal cancer cell lines [26,29,30,46]. The GI_50_ concentration for ursolic acid is 1.2–11.2 µM in renal cancer cell lines RXF393, SN12C and TK-10, with similar results observed in breast (MCF-7; 1.4–1.9 µM), colon (HCT116; 1.5–3.5 µM) and lung (NCI-H322M; 1.2–9.8 µM) cancer cell lines when cell density is measured following a 48 h treatment [29,30]. The cell density of lung (NCI-H460; 20.0 µg/mL) and cervical (ME180; 30.0 µg/mL) cancer cell lines are similarly susceptible to cranberry proanthocyanidin treatment, while breast (MCF-7), melanoma (M-14), leukemia (K562) and prostate (PC3) cancer cell lines have GI_50_ values greater than 70.0 µg/mL [26]. An organic soluble cranberry extract is effective at reducing cell density of two oral cancer cells lines, CAL27 and SCC25, at fairly similar GI_50_ concentrations, 40.0 µg/mL and 70.0 µg/mL, respectively [46]. While cell density experiments support a reduction in confluency following treatment with cranberry derivatives, these assays do not take metabolic activity, functional enzyme processes or mechanisms associated with cell death into consideration.

Extending beyond simple density studies, multiple cranberry derived extracts and constituents significantly inhibit the viability of cancer cell lines. In breast cancer cell lines, an organic-soluble cranberry extract, cranberry juice extract, a flavonoid rich fraction (with and without glycosides), quercetin and ursolic acid are all effective at decreasing the viability of MCF-7 and MDA-MB-435 cells [23,24,25,27,28]. The GI_50_ concentration of a combination flavonoid and glycoside fraction (FG; 23.9 µM) is the most efficacious against MCF-7 cells [27]. Several cranberry extracts inhibit the viability of colon cancer cell lines including an organic-soluble cranberry extract, cranberry juice extract, cranberry proanthocyanidins, a flavonoid rich fraction, a total polyphenolic fraction and ursolic acid [24,25,32,33,35,36]. Cranberry proanthocyanidins and ursolic acid are most effective at inhibiting the viability of HCT116 colon cancer cells, with GI_50_ concentrations of 25 µg/mL for both constituents [35]. LS-513 colon cancer cells are particularly susceptible to viability reductions following exposure to a total polyphenolic fraction (3.8–92.9 µg/mL) and anthocyanins (4.3–75.5 µg/mL) when compared to a cranberry juice extract (38.11–113.0 µg/mL) [32]. Cranberry proanthocyanidins also decrease the viability of neuroblastoma, esophageal adenocarcinoma and ovarian cancer cells [40,41,44,45,48,49]. All four neuroblastoma cancer cell lines (IMR-32, SH-Sy5Y, SK-N-SH and SMS-KCNR) show significant reductions in viability when treated with 12.5–25.0 µg/mL of cranberry proanthocyanidins [44,45]. In comparison, a significant reduction in viability of esophageal adenocarcinoma and ovarian cancer cells is observed with 25.0–50.0 µg/mL and 50.0–200.0 µg/mL cranberry proanthocyanidins, respectively [40,41,45,48,49]. Reductions in viability of glioblastoma and melanoma cancer cell lines occur following treatment with a flavonoid rich extract, with the GI_50_ for U87 glioblastoma cells about half of the GI_50_ for SK-MEL5 melanoma cells after a 96h treatment [28]. A significant decrease in viability is observed for HepG2 liver cancer cells when treated for 4 days with a cranberry flavonoid glycoside extract (GI_50_ = 49.2 µM), quercetin (GI_50_ = 40.9 µM) or ursolic acid (GI_50_ = 87.4 µM); where quercetin was the most effective of the three constituents [27]. Lastly, NCI-H460 cells are more susceptible to a lower dose of cranberry proanthocyanidins (50.0–100.0 µg/mL) than DMS114 cells treated with a flavonoid rich extract; however, both extracts are capable of significantly reducing cell viability of lung cancer cell lines [28,37].

A single study utilized non-dialyzable material isolated from cranberry juice reporting significant reductions in the viability of Rev-T-2-6 lymphoma cells following a 48h treatment [43]. The non-dialyzable material contains high molecular weight polyphenolic compounds, likely containing both proanthocyanidins and smaller quantities of anthocyanins [54]; however, structural characterization was not conducted due to the inability to hydrolyze the high molecular weight components into smaller oligomeric components for MALDI analysis [43]. Cancer cell lines originating from the oral cavity are susceptible to reduced viability following treatment with cranberry extract (50.0–180.6 µg/mL), cranberry juice extract (150.0 µg/mL) and a total polyphenolic fraction (200.0 µg/mL) with cranberry extract (50.0 µg/mL) treatment of CAL27 cells having the lowest GI_50_ [24,33,47]. Ten studies have documented the effectiveness of a cranberry extract, cranberry juice extract, a flavonoid rich extract and cranberry proanthocyanidins at significantly reducing the viability of prostate cancer cells [24,25,26,28,33,36,45,50,51,52]. The most efficacious constituent appears to be a cranberry proanthocyanidin extract, which inhibits the viability of RWPE-1 and RWPE-2 prostate cancer cell lines with a GI_50_ of approximately 6.5 µg/mL [33]. In contrast, prostate cancer cells are not particularly sensitive to inhibition by a flavonoid rich extract as the GI_50_ concentration for DU-145 (234.0 µg/mL) cancer cells is comparatively 1.6–3.0 fold higher compared to the GI_50_ for glioblastoma (77.0 µg/mL), melanoma (147.0 µg/mL) and breast (147.0–212.0 µg/mL) cancer cell lines [28]. Finally, two studies report cranberry and cranberry juice extract decrease the viability of stomach cancer cell lines AGS and SGC-7901 [25,53].

The majority of in vitro studies have characterized the ability of cranberry derived extracts and constituents to inhibit cancer cell line density and viability. Cranberry derived proanthocyanidins and ursolic acid tend to have lower GI_50_ values compared to the organic-soluble cranberry extracts and total phenolic fractions. It is difficult to make comparisons to the results observed for the cranberry juice extracts because concentrations are reported as µL/mL and may vary widely in concentrations of inhibitory constituents based on the starting product [25,32,47]. Furthermore, direct comparisons for specific constituents is challenging due to a lack of protocol standardization for the generation and isolation of any cranberry derived extracts or constituents. Taken together, these data show that cranberry derivatives are capable of inhibiting the viability of 31 cancer cell lines.

### 3.2. Modulation of Cell Proliferation and Cell Cycle Processes by Cranberry Constituents

The ability of cranberry derived extracts and constituents to modulate cell proliferation and cell cycle kinetics is highlighted by studies conducted in nine different target organs. Cell proliferation, as measured by BrdU incorporation, is decreased in colon, lung, ovarian and OE33 esophageal cancer cell lines following treatment with 25.0–125.0 µg/mL cranberry proanthocyanidins [37,39,45,49]. Additional esophageal adenocarcinoma cells treated with cranberry proanthocyanidins responded with an S-phase delay [39], potentially linked to induction of cell death via necrosis. A decrease in cell proliferation of stomach cancer cells is also noted following treatment with a cranberry extract but at a much higher concentration of 10.0 mg/mL [53].

Ten studies present data for modulation of cell cycle progression by cranberry derivatives. In breast cancer cells, an organic-soluble cranberry extract and a flavonoid rich extract induce G_1_ and G_2_-M cell cycle arrest, respectively [23,28]. In esophageal adenocarcinoma cells, cranberry proanthocyanidins (50.0–100.0 µg/mL) induce G_2_-M cell cycle arrest in acid-sensitive JHEsoAd1 and OE33 cells as well as in acid-resistant OE19 cells [39]. However, cranberry proanthocyanidins induce a significant S-phase delay in acid-resistant OE19 cells. In lung cancer cells, cranberry proanthocyanidins (50.0 µg/mL) induce a G_1_ cell cycle arrest [37]. Cranberry proanthocyanidins induce a G_2_-M cell cycle arrest in neuroblastoma (20.0 µg/mL) and ovarian (75.0–100.0 µg/mL) cancer cells [44,48,49]. Organic soluble cranberry extracts (25.0 µg/mL) and cranberry juice extracts (25.0 µL/mL) are both effective at inducing G_1_ cell cycle arrest in prostate cancer cells [25,50]. Finally, cranberry proanthocyanidins are more effective than a flavonoid rich extract at inducing G_1_ cell cycle arrest in glioblastoma cells in a time and dose-dependent manner [36].

### 3.3. Cranberry Derived Extracts and Constituents Induce Cell Death Pathways

Induction of cell death pathways by cranberry constituents has been investigated in nine target organs. An organic soluble cranberry extract induces apoptosis in stomach (5.0 mg/mL) and breast (15.0 mg/mL) cancer cell lines at high concentrations, whereas cancer cell death was induced at much lower concentrations (range of 50.0–70.0 µg/mL) in SCC25 and CAL27 oral cancer cell lines [23,46,53]. Treatment of esophageal adenocarcinoma, lung, colon, glioblastoma, neuroblastoma and ovarian cancer cells with cranberry proanthocyanidins results in apoptosis induction [35,36,37,39,42,44,48,49]. Ursolic acid (25.0 µg/mL) induces apoptosis in colon cancer cells and a flavonoid rich extract (100.0–400.0 µg/mL) induces late apoptosis in glioblastoma cells [28,35]. Finally, a flavonoid rich extract (200.0–400.0 µg/mL) from the cranberry is also responsible for induction of apoptosis in MDA-MB-435 cancer cells [28].

In esophageal adenocarcinoma cells, the primary form of cell death appears linked to an acid sensitive or acid resistant phenotype. Specifically, treatment with an equimolar concentration of five bile salts (taurocholic, glycocholic, glycodeoxycholic, glycochenodeoxycholic and deoxycholic acids) in acidified medium (pH 4.0) induces rapid apoptosis of esophageal adenocarcinoma cells except in the case of constitutively resistant OE19 cells were exposure to an acidified bile salt mixture has little impact on cell viability. In acid sensitive JHEsoAD1 and OE33 cell lines, cranberry proanthocyanidins induce autophagy through inactivation of Phosphoinositide 3-kinase /Protein Kinase B/mechanistic Target of Rapamycin (PI3K/AKT/mTOR) signaling pathways, but with low levels of apoptosis [39,40]. Conversely, intrinsically acid resistant OE19 cells die mainly through necrosis following treatment with cranberry proanthocyanidins. Acid sensitive JHEsoAd1 cells can be pushed to acid resistance following repeated exposure to an acidified bile cocktail resulting in a switch from autophagic to necrotic cell death [39]. The concentration of cranberry proanthocyanidins necessary to inhibit the viability of acid-resistant cells through necrosis is similar to the concentration inducing autophagy in acid-sensitive cells [39]. It is important to note that autophagy induction in JHEsoAD1 cells is a pro-death mechanism and not a pro-survival response [40,41].

In contrast to esophageal adenocarcinoma cells, the primary form of cell death induced by cranberry proanthocyanidins in neuroblastoma cells is apoptosis [44]. Interestingly, treatment of SMS-KCNR neuroblastoma (25.0 µg/mL) cancer cells for 18 h with cranberry proanthocyanidins also resulted in inactivation of PI3K/AKT/mTOR signaling pathways and at concentrations lower than those necessary for similar effects in esophageal adenocarcinoma (50.0 µg/mL) cancer cells [39,44]. The caspase apoptotic machinery in esophageal adenocarcinoma cells is expressed at low basal levels, which may explain why acid sensitive cells die primarily through autophagy [39]. Furthermore, treatment of acid sensitive esophageal adenocarcinoma cell lines with cranberry proanthocyanidins results in cell death through Beclin-1 independent autophagy induction and is largely caspase-independent despite low levels of apoptosis [39,40]. Conversely, western blot data from untreated neuroblastoma cells show that the apoptotic machinery is present [45]. Thus, cranberry proanthocyanidins modulate cell death via inactivation of the PI3K/AKT/mTOR signaling axis, but may be dependent on available cell death machinery. A decrease in signaling through the AKT pathway is also observed in SKOV-3 ovarian cancer cells treated with cranberry proanthocyanidins [49]. Furthermore, cranberry proanthocyanidins (25.0 µg/mL) increase mitogen-activated protein kinase (MAPK) signaling and decrease PI3K/AKT signaling in prostate cancer cells, suggesting a common cell death mechanism with esophageal adenocarcinoma, ovarian and neuroblastoma cancer cells [52].

### 3.4. Modulation of Oxidative Status by Cranberries

Consistent with the antioxidant properties of cranberry and cranberry derived extracts in human trials for cardiovascular disease, markers of reactive oxygen species (ROS) decrease in a number of cranberry treated cancer cell lines [15,18,21]. Specifically, malondialdehyde lipid peroxidation levels decrease in colon cancer lines following treatment with a total polyphenolic fraction (250.0 µg/mL) for 24 h [31]. An organic soluble cranberry extract and cranberry juice extract decrease malondialdehyde lipid peroxidation levels in HepG2 liver cancer cells [22]. Furthermore, an organic soluble cranberry extract and cranberry juice extract increase reduced glutathione levels and decrease glutathione peroxidase activity in HepG2 cells, respectively [22]. Total ROS levels also decrease in HepG2 liver cancer cells following treatment with an organic soluble cranberry extract (0.5 µg/mL) and cranberry juice extract (25.0 µg/mL) for 20 h [22].

Conversely, increases in ROS are reported in select cancer cell lines treated with cranberry derivatives. Specifically, cranberry proanthocyanidins increase ROS levels in esophageal adenocarcinoma (100.0 µg/mL), neuroblastoma (20.0 µg/mL) and ovarian (75.0 µg/mL) cancer cell lines [38,44,49]. The increase in JHEsoAD1 esophageal adenocarcinoma cells was partially due to increases in hydrogen peroxide levels [38]. While these data may seem counterintuitive, several drugs including cisplatin, cyclophosphamide and fenretinide used to treat cancer rely on the production of ROS as a mechanism to inhibit cancer cell viability [55,56]. Interestingly, ROS levels decrease in premalignant Barrett’s esophageal CP-C cells following treatment with cranberry proanthocyanidins, also resulting in cell death induction [38,57,58]. Given the differences observed between ROS production in premalignant Barrett’s and esophageal adenocarcinoma cells following treatment with cranberry proanthocyanidins, it is possible that basal oxidative stress levels or stage of carcinogenesis may inform functional cell death machinery and cell fate. The mechanism of cell death for premalignant esophageal cells treated with cranberry derived constituents remains to be investigated. However, immortalized “normal” Het1A esophageal cells are less susceptible to C-PAC induced cell death compared to esophageal adenocarcinoma and premalignant Barrett’s esophageal cell lines when treated with the same cranberry proanthocyanidin fraction at equivalent concentrations [40].

### 3.5. Additional Biological Processes Modulated by Cranberry Derived Extracts and Constituents

Modulation of pro-inflammatory markers, cellular adhesion, matrix metalloprotease activity and invasion of the extracellular matrix are the remaining biological processes that have been examined in cancer cell lines treated with cranberry based derivatives. Specifically, TNFα and IL-6 levels decrease following treatment with a total cranberry polyphenolic fraction (250.0 µg/mL) in colon cancer cells [31,34]. A soluble cranberry extract (50.0 µg/mL) reduces the expression of cyclooxygenase-2 (COX-2), an enzyme responsible for conversion of arachidonic acid into prostaglandins thus mitigating downstream inflammatory responses [34]. The ability of oral cancer cells to settle, adhere and establish colonies is inhibited by an organic soluble cranberry extract in a dose dependent manner [46]. In prostate cancer cells, cranberry proanthocyanidins (250.0 µg/mL) decrease matrix metalloprotease activity preventing cleavage of extracellular matrix proteins and migration of cancer cells [52]. Finally, a non-dialyzable material extract isolated from cranberry juice (50.0 µg/mL) is capable of decreasing invasion of Rev-2-T-6 lymphoma cells [43]. While these final studies describe distinctly different processes, they provide additional mechanistic information for how cranberry derived extracts and constituents modulate cancer cells in vitro and provide a foundation to support additional in vivo investigations.

### 3.6. In Vitro Summary

Collectively, data from the in vitro studies support that cranberries successfully inhibit three hallmarks of cancer: resisting cell death, sustaining proliferative signaling and activating invasion and metastasis [59]. These studies provide the preclinical basis for cancer inhibitory investigations of cranberry derived constituents utilizing in vivo models and may inform future clinical trials in high risk human cohorts. Protocol standardization for extraction and isolation of constituents from cranberries will be vital for reproducibility of data and for development of standardized products for use in human clinical trials. Cranberry proanthocyanidins appear to be among the most potent cranberry derived constituents based upon the completed in vitro research evaluating a large panel of cancer cell lines and multiple inhibitory mechanisms. Low concentrations of cranberry derived ursolic acid also favorably impacts cancer cell viability and induces apoptosis, but additional signaling networks have yet to be assessed. However, it is important to recognize that both the starting cranberry product, processing and extraction methods differed greatly across the summarized studies making direct comparisons difficult.

It is interesting to note that included in the in vitro studies is the evaluation of cranberry derived constituents in MDA-MB-435 (misidentified as breast) and M14 melanoma cancer cell lines. Controversy regarding the origin of these two cancer cell lines has been debated since 2003 when microarray studies suggested that the MDA-MB-435 cell line was not of breast origin [60]. Through extensive genetic and expression analyses, it has been established that the MDA-MB-435 cell line is of melanoma origin and is very likely a clone of the M14 melanoma cancer cell line [61]. This finding has broader implications for the scientific community, but herein expands the inhibitory effects of cranberries to include inhibition of melanoma cancer cell viability, modulation of cell cycle kinetics and provides mechanistic insight into mode of cell death induction [25,28,29].

Additional research is necessary for improved understanding of the molecular mechanisms by which cranberry derivatives inhibit cancer cells in vitro including to what extent reactive oxygen species play a role in cell death induction and whether downregulation and inactivation of the PI3K/AKT/mTOR pathway is a common mechanism. The latter is of particular importance for the potential development of neoadjuvant applications combining therapeutic drugs with natural inhibitors [62]. Natural products including quercetin, curcumin, resveratrol and lycopene reportedly impact multiple cancer-associated processes, with clinical trial results supporting the efficacy of lycopene at reducing colon cancer tumor size [63]. In addition, Singh et al. found that cranberry proanthocyanidins acted synergistically with paraplatin to inhibit SKOV-3 ovarian cancer cell viability and proliferation [45]. Importantly, pretreatment of SKOV-3 ovarian cancer cells with cranberry proanthocyanidins significantly reduced the IC_50_ following paraplatin treatment. Utilization of natural products in conjunction with chemotherapy drugs may also be useful in treating patients who have developed resistance to chemotherapeutic drugs [64]. Finally, in esophageal adenocarcinoma JHEsoAD1 cells, pharmacologic inhibition of mTOR using cranberry proanthocyanidins or rapamycin induces autophagy resulting in cell death and survival, respectively [40]. Therefore, understanding the mechanisms of cancer inhibition by cranberry constituents is critical for evaluation of cranberries in human clinical trials with a cancer prevention focus or possibility for use in combination with chemotherapeutic agents.

## 4. In Vivo Inhibition of Cancer Using Cranberry Products

There are only nine published studies assessing the preclinical efficacy of cranberry products in animal models. These studies are summarized in Table 2 and will be reviewed by target organ in the following section.

### 4.1. Cranberry Juice Concentrate and Bladder Cancer

Despite extensive research focused on cranberries and improved urinary tract health, including prevention of urinary tract infections, there is little research on cancer inhibition in vivo. Prasain et al. performed the only in vivo investigation to assess the efficacy of cranberries as inhibitors of bladder cancer [65]. Bladder cancer was induced in female Fischer-F344 rats following administration of *N*-butyl-*N*-(4-hydroxybutyl)-nitrosamine twice a week for eight weeks. Starting one week after the final carcinogen treatment, rats were gavaged daily for six months with cranberry juice concentrate (0.5 mL/rat or 1.0 mL/rat) or water. The cranberry juice concentrate utilized in this study was made available from Ocean Spray Cranberries, Inc. (City, US State, USA) and described to contain 9.57 ± 0.5 mg vanillic acid equivalent/mL of total phenolics. At the end of the six month study, bladder lesions were weighed showing that administration of high dose cranberry juice concentrate (1.0 mL/rat/day) reduced bladder tumor weight by 31%. Both doses of cranberry juice concentrate resulted in a decrease in urinary bladder tumor weight, but only the higher volume of cranberry juice concentrate (1.0 mL/rat/day) resulted in a 38% reduction in cancerous lesion formation in the bladder [65]. These data are promising supporting that an orally administered cranberry juice concentrate is non-toxic when delivered over a period of six months and that a behaviorally achievable concentration significantly inhibits progression events resulting in reduced bladder cancer [15,68].

### 4.2. Colon Cancer and Cranberries

Two studies have investigated the ability of cranberry products to reduce or inhibit colon carcinogenesis. In the first study, male F344 rats were administered either 20% cranberry juice or water ad libitum for 15 weeks with two weeks of azoxymethane (AOM) administration in weeks 4 and 5 to induce aberrant crypt foci (ACF) and colon cancer [66]. There was a 77% reduction in the number of AOM-induced ACF in rats administered cranberry juice, with reductions in both the proximal and distal colon. Finally, animals had significantly higher levels of liver glutathione-*S*-transferase activity compared to untreated controls supporting that cranberry juice may activate cell protection mechanisms against oxidative stress in the context of colon cancer.

In a study performed by Ferguson et al., the colon carcinoma cell line HT-29 was utilized to establish xenografts in female NCR NU/NU mice [36]. Specifically, HT-29 (5.0 × 10^6^) cells were subcutaneously injected into the right flank of mice and tumors were monitored every other day. Cranberry proanthocyanidins (100.0 mg/kg) were administered intraperitoneally 2–3 times per week for a total of ten injections over 24 days. Mice treated with cranberry proanthocyanidins had significantly lower body weights early in the study but weights returned to normal in the last five days of the study raising the question of initial toxicity at the administered concentration. Importantly, cranberry proanthocyanidins significantly inhibited the growth of HT-29 tumor xenografts in mice.

The final colon related study utilized a dextran sodium sulfated (DSS)-induced mouse model of colitis [67]. DSS-induced colitis is widely accepted as an animal model of irritable bowel syndrome, a condition linked to elevated risk for colorectal cancer development [69]. In this study, male Balb/C mice were fed a diet containing either cranberry extract (0.1% or 1%) or 1.5% dried whole cranberry powder in the food *ad libitum*. On the third and sixth weeks of the study, the water was replaced with 1% w/v of DSS solution for one week. Animals fed either the cranberry extract or the dried whole cranberry powder had a significantly lower disease activity index (more normal stool consistency and decreased blood in fecal samples) compared to controls for both DSS treatment periods. Furthermore, 1% cranberry extract and the 1.5% dried whole cranberry powder diets delayed the onset of colitis. In addition, inflammatory markers including TNFα (significant in all cranberry treatments) and IL-1β (1.5% dried whole cranberry powder only) were decreased in colonic tissues, suggesting that cranberry derived extracts and powders exert an anti-inflammatory role in vivo.

These data parallel in vitro data for cell death induction, antioxidant effects and anti-inflammatory properties of cranberries and cranberry derived constituents. In the studies by Boateng et al. and Xiao et al. [66,67], the cranberry products were administered throughout the entire bioassay and although encouraging consideration in future studies should be given to discerning whether cranberry products possess both anti-initiation as well as anti-promotion/progression properties in vivo. As colon cancer and a western diet are reportedly linked, regular inclusion of cranberries may impart cancer preventative advantages [70].

### 4.3. Cranberry Proanthocyanidins and Esophageal Adenocarcinoma

The cancer inhibitory potential of cranberry proanthocyanidins against esophageal adenocarcinoma was investigated by Kresty et al. using murine xenografts [39]. Acid-resistant OE19 (1.25 × 10^6^) cells were subcutaneously implanted in each flank of twelve male NU/NU athymic mice and tumors were established (150 mm^3^) prior to initiation of cranberry proanthocyanidin treatment. Five days after cell injection, mice were randomized for treatment with cranberry proanthocyanidins (250.0 µg/mouse) or vehicle by oral gavage six days a week. There was a significant difference in tumor volume between vehicle and cranberry proanthocyanidin treated mice by day 12, with the study ending on day 19 due to large tumor size among vehicle treated controls. Mean tumor volume in cranberry proanthocyanidin treated mice was reduced by 67.6% and showed reduced inflammation compared to tumors isolated from control mice. Importantly, tumor lysates from cranberry proanthocyanidin treated mice showed inactivation of PI3K/AKT/mTOR signaling as was observed in vitro utilizing a panel of esophageal adenocarcinoma cell lines [39]. Interestingly, acid-sensitive JHAD1 and OE33 cells were not able to form tumors in this xenograft model, suggesting that the OE19 cells are phenotypically more aggressive and that acid-resistance may support tumor development.

### 4.4. Glioblastoma and Cranberry Derived Constituents

The efficacy of cranberry proanthocyanidins and a flavonoid rich cranberry extract for prevention of glioblastoma tumors was evaluated by Ferguson et al. in a murine xenograft model [36]. U87 (1.0 × 10^6^) glioblastoma multiforme cancer cells were injected subcutaneously into the right flank of female NCR NU/NU mice and tumors were monitored every other day. Mice were administered cranberry proanthocyanidins (100.0 mg/kg body weight) or a flavonoid rich extract (250.0 mg/kg body weight) intraperitoneally 2–3 times per week for a total of ten injections. Both cranberry fractions were able to slow tumor growth by up to 40%. These data suggest that cranberry derived extracts may be effective against glioblastoma muliforme but the mechanisms remain to be characterized.

### 4.5. Non-Dialyzable Material from Cranberry Juice and Lymphoma

Inhibition of lymphoma xenograft growth by cranberry juice in a murine model was investigated by Hochman et al. [43]. Rev-2-T-6 (5.0 × 10^6^) lymphoma cancer cells were injected into female Balb/C mice and animals were subsequently treated with cranberry juice constituents (130.0 mg/kg body weight) intraperitoneally every other day for two weeks. The treatment consisted of non-dialyzable material from cranberry juice with a molecular weight range of 12–30 kDa. Mice treated with non-dialyzable material did not form tumors with 80% of control mice developing lymphomas. This study is positive for the prevention of lymphoma by cranberry juice but due to study design the results fail to discriminate between failed xenograft implementation and prevention of tumor growth.

### 4.6. Prostate Cancer and Cranberry Proanthocyanidins

Utilizing a murine xenograft model with prostate cancer cells, Ferguson et al. showed that cranberry proanthocyanidins inhibit tumors in vivo [36]. DU-145 (4.0 × 10^6^) prostate cancer cells were subcutaneously implanted in the right flank of female NCR NU/NU mice. On day 2, mice were administered cranberry proanthocyanidins (100.0 mg/kg body weight) intraperitoneally every 2 or 3 days for a total of 10 injections. Cranberry proanthocyanidins significantly inhibited the growth of tumors compared to control mice and two of the mice had tumor regression by 108 days post implant. These data are encouraging for the inhibition of prostate tumor development by cranberry proanthocyanidins, but additional experiments are necessary for investigating the mechanism of tumor regression.

### 4.7. Whole Cranberry Extract and Stomach Cancer

Efficacy of a whole cranberry extract inhibiting stomach cancer tumor growth was evaluated in a murine xenograft model developed by Liu et al. [53]. Balb/C NU/NU mice were subcutaneously injected with SGC-7901 (5.0 × 10^6^) gastric adenocarcinoma cells in the right flank region. Prior to implantation, SGC-7901 cells were pretreated with a whole cranberry extract (0–40.0 mg/mL) for 48 h. Tumors in control mice developed by the tenth day of the experiment with a delay of 3–7 days for cells pretreated with 5.0–20.0 mg/mL of cranberry extract. Xenograft tumors did not develop from SGC-7901 cells pretreated with 40.0 mg/mL cranberry extract but in vitro data presented in the same study supported that these cells were likely undergoing high levels of apoptosis with reduced viability. Therefore, additional studies will be necessary to determine how a whole cranberry extract inhibits the growth of stomach cancer in animal models, including those which model gastric carcinogenesis as a result of chronic *Helicobacter pylori* infection [71].

### 4.8. In Vivo Summary

The in vivo studies described here provide preliminary evidence for the preclinical efficacy of multiple cranberry derived extracts against seven cancer targets. Except for the studies in esophageal adenocarcinoma and colon cancer, the majority of completed in vivo studies reporting inhibition of tumor development or growth, fail to include further mechanistic assessments. Of the nine in vivo studies, three studies used carcinogens or chemicals to induce cancer in animal models. In the bladder, delivery of a cranberry juice concentrate by gavage following carcinogen treatment supports anti-promotion/progression effects of cranberries against chemically-induced bladder cancer. Two studies in the colon assessed the efficacy of cranberry juice, cranberry extract powder and a dried whole cranberry powder in a full carcinogenesis schematic, where dietary administration of cranberries began prior to carcinogen initiation and continued throughout, after carcinogen or chemical treatment was completed. In regard to mode of delivery, four of the in vivo studies delivered the cranberry product by orally, either in water, diet or gavage with efficacy suggesting that the compounds or their metabolites hold promise as orally bioavailable cancer inhibitors. Administration of cranberry products via intraperitoneal injection also showed cancer inhibitory efficacy in four in vivo studies, but as a mode of delivery is less relevant for primary or secondary cancer prevention efforts in human cohorts. Overall, the in vivo results expand upon in vitro observations and importantly support that long-term administration of cranberry products is well tolerated and cancer inhibitory in various animal models. However, additional research focused on bioavailability, metabolic fate and additional cancer inhibitory mechanisms of cranberry products is warranted for informing clinical focused cancer prevention efforts.

To date only a few human studies have characterized cranberry metabolites in plasma or urine and often these studies are limited to quantifying molecules that have previously been identified [15,16,72,73,74,75]. A recent study by McKay et al. reported that flavonoids, phenolic acids and proanthocyanidins can be detected in the urine or plasma of individuals who consumed a 54% cranberry juice cocktail [15]. Recent advances in standards used for identification and quantification of cranberry metabolites has resulted in the identification of 60 metabolites from the urine and plasma of healthy men after consumption of a cranberry juice cocktail that contained 787 mg of polyphenols [68]. The ability to detect and quantitate proanthocyanidins in the urine and plasma is not consistent from study to study, but this should improve with the recent development of a new cranberry proanthocyanidin standard that more closely reflects the structural heterogeneity of proanthocyanidins present in fresh cranberries [76]. Cranberry proanthocyanidins are large, complex molecules with recent data supporting that the intestinal microbiome is responsible for the metabolism of cranberry proanthocyanidins into smaller active metabolites [68]. Additional research will be necessary to assess the bioavailability and metabolism of cranberries in humans and recent advances in standards and the radiolabeling of cranberry products will provide new tools to aid investigations.

## 5. Conclusions

Evaluation of cranberries and cranberry derived constituents in preclinical in vitro and in vivo studies evaluating cancer inhibition is key for the future development of cranberry-based interventions in high-risk human cohorts. The data presented in this review strongly support the anti-proliferative and pro-death capacities of cranberries in a multitude of cancer cell lines and select in vivo models including xenograft and chemically induced cancer models. The precise cancer inhibitory mechanisms associated with cranberries in specific targets are still be elucidated, but preclinical studies utilizing cranberry proanthocyanidins show inactivation of the PI3K/AKT/mTOR pathways and modulation of MAPK signaling in esophageal, neuroblastoma, ovarian and prostate cancer cells and in esophageal xenografts [39,44,49,52]. Moreover, cranberry proanthocyanidins have recently been shown to induce autophagic markers in vitro and in vivo [39], suggesting an alternative mode of cell death induction and tumor inhibition that requires further evaluation in additional cancer targets. A recent study published by The Cancer Genome Atlas Network showed that a large number of genetic alterations were shared across 279 patients with head and neck squamous cell carcinomas including activating mutations in *PIK3CA*, the gene encoding the catalytic subunit of phosphatidylinositol 3-kinase (PI3K) [77]. Cranberry proanthocyanidins are also known to mitigate inflammatory responses of oral epithelial cells and to inhibit oral biofilm formation [12,78]; thus, cranberry derived constituents may be particularly efficacious inhibitors targeting oral premalignancy. Researchers should continue to define the mechanisms of cancer inhibition in vitro and in vivo with the goal of informing mechanistically driven human clinical trials. It should be noted that the efficacy of natural products in head and neck, esophageal and colon cancers has been demonstrated by black raspberries [79]. The use of cranberries and cranberry derived constituents in cancer prevention is at an early stage. Still, results are highly promising considering positive preclinical results following treatment at relatively low, behaviorally achievable, concentrations when administered in a drink formulation, consumed as food or as a supplement. A recent study by Marette *et al.* reported cranberry polyphenols protect from diet-induced obesity, insulin resistance and intestinal inflammation [80]. The latter research findings were associated with a shift in fecal microbiome profiles and although cancer was not an outcome under evaluation, further assessments of cranberries targeting obesity-linked cancers seems logical. In conclusion, additional research focused on issues of metabolism, bioavailability, pharmacokinetics, pharmacodynamics, active fractionation, optimum dose, formulation, routes of delivery and duration are required to inform the cancer preventive utility of cranberries in high risk human cohorts.

## Figures and Tables

**Table 1:** Summary of preclinical in vitro evaluations of cranberries or cranberry derived constituents as cancer inhibitors.

Target	Cell Line(s)	Cranberry Constituent	In Vitro Results [Reference(s)]
Breast	MCF-7	CE	↑ apoptosis [23]; ↑ G_1_ cell cycle arrest [23]
			↓ cell viability [23,24]
		CJE	↓ cell viability [25]
		C-PAC	↓ cell density [26]
		FG	↓ cell viability [27]
		Fr6	↓ cell viability [28]
		Q	↓ cell viability [27]
		UA	↓ cell density [29,30]; ↓ cell viability [27]
	MDA-MB-435*	CJE	↓ cell viability [25]
		Fr6	↑ apoptosis [28]; ↑ G_2_-M cell cycle arrest [28]
			↓ cell viability [28]
		UA	↓ cell density [29]
Cervix	ME180	C-PAC	↓ cell density [26]
		UA	↓ cell density [30]
Colon	Caco-2	CJE	↓ cell viability [25]
		TP	↓ lipid peroxidation [31]
			↓ pro-inflammatory markers TNFα and IL-6 [31]
	HT-29	ANTHO	↓ cell viability [32]
		CE	↓ cell viability [24,33]
			↓ pro-inflammatory marker COX-2 [34]
		C-PAC	↑ apoptosis [35]; ↓ cell density [26]
			↓ cell viability [36]
		CJE	↓ cell viability [32]
		Fr6	↓ cell viability [36]
		TP	↓ cell viability [32]
		UA	↑ apoptosis [35]; ↓ cell density [29,30]
			↓ cell viability [35]
	HCT116	CE	↓ cell viability [33]
		C-PAC	↑ apoptosis [35]; ↓ cell viability [35]
		UA	↑ apoptosis [35]; ↓ cell density [29]
			↓ cell viability [35]
	LS-513	ANTHO	↓ cell viability [32]
		CJE	↓ cell viability [32]
		TP	↓ cell viability [32]
	SW460	TP	↓ cell viability [33]
	SW620	TP	↓ cell viability [24]
		C-PAC	↓ cell proliferation [37]
Esophagus	CP-C	C-PAC	↓ total reactive oxygen species [38]
	JHEsoAD1	C-PAC	↑ autophagy in acid-sensitive cells, pro-death [39,40]
			↑ necrosis in acid-resistant cells [39]
			↑ G_2_-M cell cycle arrest [39]
			↑ total reactive oxygen species [38]
			↑ hydrogen peroxide levels [38]
			↓ cell viability [40,41]
			↓ PI3K/AKT/mTOR signaling [39]
	OE33	C-PAC	↑ autophagy in acid-sensitive cells [39]
			↑ low levels of apoptosis [39]↑ G_2_-M cell cycle arrest [39]
			↓ cell proliferation [39]
			↑ total reactive oxygen species [38]
			↓ PI3K/AKT/mTOR signaling [39]
	OE19	C-PAC	↑ necrosis in acid-resistant cells [39]
			↑ G_2_-M cell cycle arrest with significant S-phase delay [39]
			↑ total reactive oxygen species [38]
			↑ hydrogen peroxide levels [38]
			↓ PI3K/AKT/mTOR signaling [39]
			↓ cell viability [40,41]
Glioblastoma	SF295	UA	↓ cell density [29]
	U87	C-PAC	↑ apoptosis [36]; ↑ G_1_ cell cycle arrest [36]
			↓ cell viability [36]
		Fr6	↑ apoptosis [36]; ↑ G_1_ cell cycle arrest [36]
			↓ cell viability [28]
Leukemia	K562	C-PAC	↓ cell density [26]
	RPMI8226	UA	↓ cell density [29]
Liver	HepG2	CE	↑ reduced glutathione levels [22]
			↓ glutathione peroxidase activity [22]
			↓ lipid peroxidation [22]
			↓ reactive oxygen species [22]
		CJE	↑ reduced glutathione levels [22]
			↓ glutathione peroxidase activity [22]
			↓ lipid peroxidation [22]
			↓ reactive oxygen species [22]
		FG	↓ cell viability [27]
		Q	↓ cell viability [27]
		UA	↓ cell viability [27]
Lung	DMS114	Fr6	↓ cell viability [28]
	NCI-H322M	UA	↓ cell density [29]
	NCI-H460	C-PAC	↑ apoptosis [37,42]; ↑ G_1_ cell cycle arrest [37]
			↓ cell density [26]; ↓ cell viability [37]
			↓ cell proliferation [37]
		UA	↓ cell density [29,30]
Lymphoma	Rev-2-T-6	NDM	↓ cell viability [43]
			↓ extracellular matrix invasion [43]
Melanoma	M14	C-PAC	↓ cell density [26]
		UA	↓ cell density [30]
	SK-MEL5	Fr6	↓ cell viability [28]
Neuroblastoma	IMR-32	C-PAC	↓ cell viability [44]
	SH-Sy5Y	C-PAC	↓ cell viability [44]
	SK-N-SH	C-PAC	↓ cell viability [44]
	SMS-KCNR	C-PAC	↑ apoptosis [44]; ↑ G_2_-M cell cycle arrest [44]
			↑ reactive oxygen species [44]
			↓ PI3K/AKT/mTOR signaling [44]
			↓ cell viability [44,45]
Oral Cavity	CAL27	CE	↑ apoptosis [46]; ↓ cell adhesion [46]
			↓ cell density [46]; ↓ cell viability [24]
		TP	↓ cell viability [33]
	HSC2	CJE	↑ reduced glutathione levels [47]
			↓ cell viability [47]
	KB	CE	↓ cell viability [24]
		TP	↓ cell viability [33]
	SCC25	CE	↑ apoptosis [46]; ↓ cell adhesion [46]
			↓ cell density [46]
Ovary	OVCAR-8	C-PAC	↑ G_2_-M cell cycle arrest [48]; ↓ cell viability [48]
	SKOV-3	C-PAC	↑ apoptosis [48,49]; ↑ G_2_-M cell cycle arrest [48,49]
			↑ reactive oxygen species [49]
			↓ AKT signaling [49]
			↓ cell proliferation [45,49]
			↓ cell viability [45,48,49]
Prostate	22Rv1	CE	↓ cell viability [33]
		TP	↓ cell viability [33]
	DU-145	CE	↑ G_1_ cell cycle arrest [50]
			↓ cell viability [50,51]
		C-PAC	↑ apoptosis [51]
			↑ MAPK signaling [52]
			↓ cell viability [26,36,51,52]
			↓ matrix metalloprotease activity [52]
			↓ PI3K/AKT signaling [52]
		Fr6	↓ cell viability [28,36]
	LNCaP	CE	↓ cell viability [24]
	PC3	CJE	↑ G_1_ cell cycle arrest [25]
			↓ cell viability [25]
		C-PAC	↓ cell density [26]
		UA	↓ cell density [30]
	RWPE-1	CE	↓ cell viability [33]
		C-PAC	↓ cell viability [33]
		TP	↓ cell viability [33]
	RWPE-2	CE	↓ cell viability [33]
		C-PAC	↓ cell viability [33]
		TP	↓ cell viability [33]
Renal	RXF393	UA	↓ cell density [29]
	SN12C	UA	↓ cell density [29]
	TK-10	UA	↓ cell density [29]
Stomach	AGS	CJE	↓ cell viability [25]
	SGC-7901	CE	↑ apoptosis [53]
			↓ cell proliferation [53]
			↓ cell viability [53]

Cranberry derived constituents are abbreviated as follows: anthocyanins (ANTHO), organic-soluble cranberry extract (CE), cranberry juice extract (CJE), cranberry proanthocyanidin-rich fraction (C-PAC), flavonoid-rich fraction 6 (Fr6), flavonoid glycosides (FG), non-dialyzable material from cranberry juice concentrate (NDM), total polyphenolic fraction (TP), quercetin (Q) or ursolic acid fraction (UA). Additional abbreviations: Phosphoinositide 3-kinase (PI3K), Protein Kinase B (AKT), mechanistic Target of Rapamycin (mTOR), mitogen-activated protein kinase (MAPK). Note: MDA-MB-435* was misidentified as a breast cancer cell line, but is now confirmed to be of melanoma origin.

**Table 2:** Summary of preclinical in vivo evaluations of cranberry products as cancer inhibitors.

Target Organ	In Vivo Models/Cranberry Product and Mode of Delivery/Results
[Reference]	
Bladder	
[65]	Nitrosamine-induced tumors in female F344 rats for eight weeks; following a one-week break, treatment with 0.5 mL/rat or 1.0 mL/rat with cranberry juice concentrate by gavage daily for six months; 31% reduction in bladder tumor weight and 38% reduction in cancerous lesion formation.
Colon	
[66]	AOM-induced ACF in male F344 rats three weeks after initiation of cranberry juice treament; ad libitum access to 20% cranberry juice in water for 15 weeks; 77% reduction in AOM-induced ACF with reductions in the proximal and distal colon versus untreated controls; significantly increased levels of liver glutathione-*S*-transferase versus controls.
[36]	HT29 (5.0 × 10^6^ cells) xenografts in female NCR NU/NU mice; treatment with cranberry proanthocyanidins (100.0 mg/kg body weight) intraperitoneally three times weekly for 24 days; significant inhibition of explant growth versus controls.
[67]	DSS induced experimental colitis in male Balb/c mice at weeks three and six; Treatment with cranberry extract powder (0.1% or 1.0%) or 1.5% freeze dried whole cranberry powder in diet ad libitum from start until ≥ six weeks; cranberry extract powder (1.0%) and 1.5% dried whole cranberry powder treatment normalized stool consistency, decreased blood in fecal samples versus controls and reduced late onset colitis; all treatments decreased serum TNFα levels.
Esophagus	
[39]	OE19 (1.25 × 10^6^ cells) xenografts in male athymic NU/NU mice; treatment with cranberry proanthocyanidins (250.0 µg/mouse) by oral gavage six days/week for 19 days; 67% decrease in mean tumor volume versus controls and treatment modulated multiple cancer signaling pathways including inactivation of the PI3K/AKT/mTOR pathway.
Glioblastoma	
[36]	U87 (1.0 × 10^6^ cells) xenografts in female NCR NU/NU mice; treatment with cranberry proanthocyanidins (100.0 mg/kg body weight) or a flavonoid rich cranberry fraction (250.0 mg/kg body weight) intraperitoneally three times a week; significant inhibition of explant growth by both fractions versus controls
Lymphoma	
[43]	Rev-2-T-6 (5.0 × 10^6^ cells) xenografts in female Balb/C mice; treatment with non-dialyzable material from cranberry juice concentrate (160.0 mg/kg body weight) intraperitoneally three times a week; significant inhibition of explant growth.
Prostate	
[36]	DU-145 (4.0 × 10^6^ cells) xenografts in female NCR NU/NU mice; treatment with cranberry proanthocyanidins (100.0 mg/kg body weight) intraperitoneally three times a week; significant inhibition of explant growth by cranberry proanthocyanidin fraction.
Stomach	
[53]	SGC-7901 (5.0 x 10^6^ cells) xenografts in Balb/c NU/NU mice; SGC-7901 cells were pre-treated with cranberry extract prior to xenograft implantation; increased tumor latency and reduced tumor size in a dose-dependent manner.

Abbreviations: azoxymethane (AOM), athymic nude mice (NCR NU/NU), dextran sodium sulfate (DSS).
